# Forever young: rejuvenating muscle satellite cells

**DOI:** 10.3389/fnagi.2015.00037

**Published:** 2015-04-21

**Authors:** Luca Madaro, Lucia Latella

**Affiliations:** ^1^Epigenetics and Regenerative Medicine, IRCCS Fondazione Santa LuciaRome, Italy; ^2^Institute of Cell Biology and Neurobiology, National Research Council of ItalyRome, Italy; ^3^Institute of Translational Pharmacology, National Research Council of ItalyRome, Italy

**Keywords:** muscle satellite cells, muscle regeneration, muscle aging, p38 mitogen-activated protein kinases, p16INK4a

## Abstract

A hallmark of aging is alteration of organismal homeostasis and progressive decline of tissue functions. Alterations of both cell intrinsic functions and regenerative environmental cues contribute to the compromised stem cell activity and reduced regenerative capability occurring in aged muscles. In this perspective, we discuss the new evidence supporting the hypothesis that skeletal muscle stem cells (MuSCs) are intrinsically defective in elderly muscles. In particular, we review three recent papers leading to identify fibroblast growth factor receptor-1, p38 mitogen-activated protein kinase, and p16INK4a as altered signaling in satellite cells from aged mice. These pathways contribute to age-related loss of MuSCs asymmetric polarization, compromised self-renewal capacity, and acquisition of pre-senescent state. The pharmacological manipulation of those networks can open novel strategies to rejuvenate MuSCs and counteract the functional decline of skeletal muscle during aging.

Extended lifespan raises the issue of handling age-related disorders, which profoundly affect the quality of life of an increasing number of people. At the physiological level, the most relevant feature of aging is the functional decline of tissue functions (Oh et al., 2014).

In particular, in the elderly, muscle mass declines progressively by means of a process named sarcopenia, making skeletal muscle one of the more compromised tissues during aging. Beyond the protein breakdown associated with the loss of sarcomeric proteins, aged muscles display compromised regenerative capacity associated with altered environmental cues (Kim and Choi, 2013; Sayer et al., 2013).

Muscle regeneration is achieved by the interplay between adult stem cells, named muscle satellite cells (MuSCs), and other cellular types (i.e., macrophages and muscle interstitial cells) that participate in the orchestration of regeneration. Muscle niche derived and systemic cues contribute to regulate muscle homeostasis and functionality (Chakkalakal et al., 2012; Bentzinger et al., 2013). Changes of those three compartments are described throughout lifetime and account for the decline of functional capacities in the elderly (Jang et al., 2011). Upon muscle injury, MuSCs that are located in a niche between the basal lamina and the sarcolemma, become activated and recapitulate myogenic differentiation to replenish damaged muscle (Collins et al., 2005; Cheung and Rando, 2013). Additionally, environmental cues finely regulate this process driving efficient muscle regeneration (Sinha et al., 2014). In order to ensure optimal performance, it is critical that several properties of MuSCs are finely regulated and coordinated. Amongst these properties are survival, self-renewal, fine-tuning between exit from quiescence and proliferative expansion, and eventually commitment toward myogenic differentiation (Bentzinger et al., 2013). All these processes are altered in the elderly leading to compromised muscle functionality.

Beyond the notion provided by parabiosis experiments that circulating systemic factors are able to restore muscle regeneration in aged mice (Conboy et al., 2005), recent evidence supports the hypothesis that MuSCs are intrinsically defective in aged muscles. These new findings open the possibility to target this stem cell compartment to counteract functional decline of muscle during aging. Here, we will provide a general comment on three breakthrough studies from Bernet et al. (2014), Sousa-Victor et al. (2014) and Cosgrove et al. (2014) discussing the relative contribution to muscle regeneration of cell-autonomous vs. cell non-autonomous factors during aging.

In their work Bernet et al. and Cosgrove et al. provide evidence that constitutive activation of the p38 MAPK in aged MuSCs leads to a decline in their self-renewal and regenerative capacity. Both groups demonstrated that partial pharmacological inhibition of p38 is sufficient to restore the ability of MuSCs to participate efficiently in muscle regeneration and to maintain the stem cell pool. Interestingly, Bernet et al. identify an alteration of the FGF-2/FGFR1 axis as a feature of aged MuSC dysfunction, as observed previously by Brack and colleagues (Chakkalakal et al., 2012). Although in the paper by Chakkalakal the authors suggest that increased activity of FGFR1 results in the disruption of MuSC quiescence in aged muscles, the Bernet study supports the hypothesis that FGF-2 increase in the aged niche is a compensatory response to the loss of function of FGFR1 activity observed in aged MuSCs. In particular, they show that while in young MuSCs the FGF2/FGFR1 axis drives asymmetric division through activation of p38 only in the committed daughter cell, in aged MuSCs this balance is altered. Indeed, the insensitivity to FGF signaling in the elderly MuSCs results in constitutive activation of p38 with loss of asymmetric polarization and impaired self-renewal capacity. Likewise, FGFR1 ligand independent, constitutive activation restores MuSC asymmetric cell division.

With elegant experiments of autologous and serial MuSC transplantation Cosgrove et al. demonstrate the intrinsic defect of elderly derived MuSCs in association with increased p38 activity. The authors demonstrate a synergistic interaction of biochemical and biophysical factors, respectively pharmacological inhibition of p38 and a hydrogel culture system, which contribute to reconstitute the proliferative capability and self-renewal as assayed by *in vitro* and *in vivo* engraftment. The effect of p38 inhibition in driving stem cell renewal was already demonstrated by Palacios et al. (2010), supporting the notion that pharmacological intervention with p38 inhibitors may support muscle regeneration. Moreover, this paper provides a useful strategy to overcome the bottleneck of *in vitro* stem cell expansion in cell therapies using specific soft biomaterial that mimics the muscle niche.

In the same month Sousa-Victor and colleagues came out with a study demonstrating that geriatric MuSCs fail to support muscle regeneration and display defective activation. Serial transplantation experiments supported the conclusion that this defect is a cell intrinsic feature of geriatric MuSCs. They identify the master regulator of senescence p16^INK4a^ as a key determinant responsible for a quiescence-senescence switch (a process named geroconversion) operating in geriatric MuSCs in coincidence with their impaired regenerative potential. Indeed, genetic inactivation of p16^INK4a^ locus was sufficient to recover the cells from the senescence-associated cell cycle arrest and restore their self-renewal capacity, leading to the reconstitution of the stem cell pool after muscle damage. The novelty of this study relies on the finding that geriatric stem cells are associated with the progressive accumulation of DNA damage and senescence-associated markers that in turn contribute to the loss of reversible quiescence mediated by p16^INK4a^. Indeed, in geriatric MuSCs, the p16^INK4a^ locus is constitutively de-repressed due to altered PRC1 complex function.

These studies demonstrate that in addition to the regenerative environment that profoundly affects the niche and stem cell function, there is another level of tissue homeostasis regulation that is intrinsic to adult stem cells. The cell autonomous functionality declines in the elderly due to de-regulated p38 signaling and accumulation of DNA damage and senescence-associated features. This evidence suggests new avenues to reverse the dysfunctional status of MuSCs from aged tissues. For instance, constitutive FGFR1 signaling can restore MuSCs asymmetric division and self-renewal, and pharmacological blockade of p38 signaling can promote MuSCs self-renewal and engraftment by silencing p16^INK4a^, thus reversing geroconversion and allowing MuSCs to support muscle regeneration (Table 1). Intriguingly, the activation of p38 signaling has been associated with senescence (Wang et al., 2002) as well as increasing levels of p16^INK4a^ (Serrano et al., 1997; Iwasa et al., 2003) in cell types other than muscle stem cells highlighting the notion that a more complex signaling network that may be context dependent controls senescence (Xu et al., 2014). The p38 signaling pathway has been demonstrated to be involved in IL-6 induced STAT3 transcriptional activation (Zauberman et al., 1999; Riebe et al., 2011). Intriguingly, the recent finding that increases in JAK-STAT signaling inhibits MuSCs function during aging further provides evidence for the pivotal role of p38 in driving muscle regeneration (Price et al., 2014; Tierney et al., 2014). Future studies should determine the molecular relationship between these new players of muscle aging—DNA damage, p38 signaling and p16^INK4a^ in order to devise treatments aimed at reversing MuSC senescence.

**Table 1:**
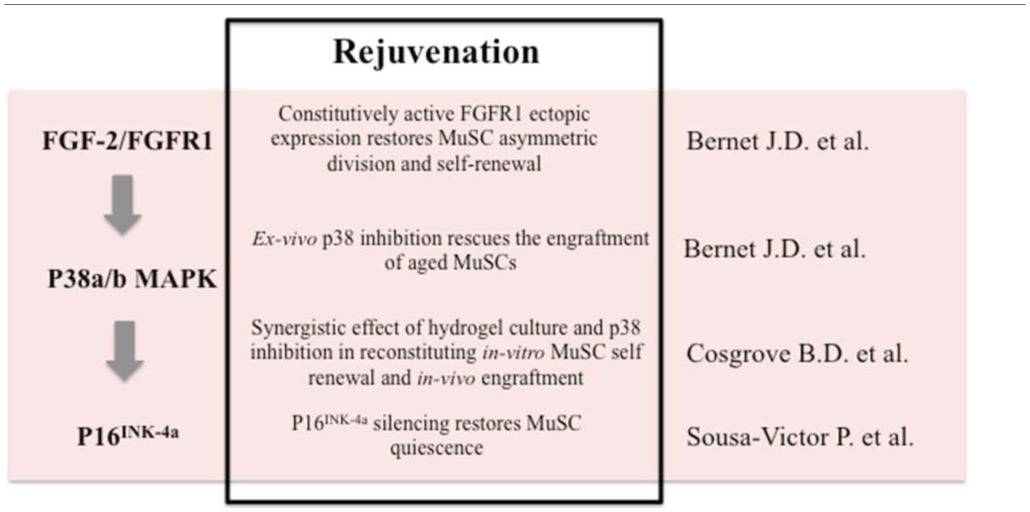
**Schematic representation of the rejuvenation strategies used in the discussed papers**.

## Conflict of interest statement

The authors declare that the research was conducted in the absence of any commercial or financial relationships that could be construed as a potential conflict of interest.
